# Evaluation of PET quantitation accuracy among multiple discovery IQ PET/CT systems via NEMA image quality test

**DOI:** 10.1186/s40658-020-00294-y

**Published:** 2020-05-12

**Authors:** Delphine Vallot, Elena De Ponti, Sabrina Morzenti, Anna Gramek, Anna Pieczonka, Gabriel Reynés Llompart, Jakub Siennicki, Paul Deak, Chiranjib Dutta, Jorge Uribe, Olivier Caselles

**Affiliations:** 1 Medical Physics Department, Institut Claudius Regaud, Toulouse, France; 2 Medical Physics Department, ASST-Monza, San Gerardo Hospital, Italy; 3 Affidea, Poznan, Poland; 4 Affidea, Wroclaw, Poland; 5 PET Unit Nuclear Medicine, IDI, Hospital U. de Bellvitge, Barcelona, Spain; 6 General Electric Healthcare, Waukesha, USA

**Keywords:** PET/CT, Multi-center harmonization, quantitation, ^18^F-FDG NEMA body phantom, ^68^Ge solid phantom

## Abstract

**Introduction:**

Quantitative imaging biomarkers are becoming usual in oncology for assessing therapy response. The harmonization of image quantitation reporting has become of utmost importance due to the multi-center trials increase. The NEMA image quality test is often considered for the evaluation of quantitation and is more accurate with a radioactive solid phantom that reduces variability. The goal of this project is to determine the level of variability among imaging centers if acquisition and imaging protocol parameters are left to the center’s preference while all other parameters are fixed including the scanner type.

**Methods:**

A NEMA-IQ phantom filled with radioactive ^68^Ge solid resin was imaged in five clinical sites throughout Europe. Sites reconstructed data with OSEM and BSREM algorithms applying the sites’ clinical parameters. Images were analyzed according with the NEMA-NU2-2012 standard using the manufacturer-provided NEMA tools to calculate contrast recovery (CR) and background variability (BV) for each sphere and the lung error (LE) estimation. In addition, a ^18^F-filled NEMA-IQ phantom was also evaluated to obtain a gauge for variability among centers when the sites were provided with identical specific instructions for acquisition and reconstruction protocol (the aggregate of data from 12 additional sites is presented).

**Results:**

The data using the ^68^Ge solid phantom showed no statistical differences among different sites, proving a very good reproducibility among the PET center models even if dispersion of data is higher with OSEM compared to BSREM. Furthermore, BSREM shows better CR and comparable BV, while LE is slightly reduced. Two centers exhibit significant differences in CR and BV values for the ^18^F NEMA NU2-2012 experiments; these outlier results are explained.

**Conclusion:**

The same PET system type from the various sites produced similar quantitative results, despite allowing each site to choose their clinical protocols with no restriction on data acquisition and reconstruction parameters. BSREM leads to lower dispersion of quantitative data among different sites. A solid radioactive phantom may be recommended to qualify the sites to perform quantitative imaging.

## Introduction

The current clinical trend is to use PET/CT as a quantitative biomarker in oncology for assessing the reliable evaluation of lesion’s uptake changes over time. The standardized uptake value (SUV) is still the standard quantitative index in positron emission tomography (PET), but new surrogates are now reported as references like response criteria in solid tumors (PERCIST) [1], metabolic active tumor volumes (MATV), or total lesion glycolysis (TLG) [2].

For all these new quantitative indexes, long-term accuracy and reproducibility are mandatory [3, 4]. Nevertheless, these parameters are known to be strongly influenced by various factors such as injected activity, uptake and acquisition time, reconstruction parameters, and lesion’s dimension and localization [5, 6]. The technological advances and design improvements in PET systems lead to higher sensitivities and are now associated with advanced reconstruction algorithms including accurate corrections applied during the iterative process (e.g., point spread function (PSF), time of flight (TOF), block sequential regularized expectation maximization (BSREM) for noise regularization). As a result, this large number of parameters lead to variations observed in quantitative measurements [7]. The need for harmonization in quantitative reporting has become of the utmost importance due to the increasing number of multi-center clinical trials. Several protocols are available to limit the differences among the scans outcome, from the patient preparation to the final report: for instance, the Uniform Protocols for Imaging in Clinical Trials (UPICT), the ResEARrch 4 Life (EARL) accreditation protocol, or the EANM FDG guidelines [7–9]. These protocols are expected to increase SUV’s absolute evaluation, but not all the sites in Europe are currently accredited by EANM.

Chauvie et al. [10] clearly demonstrated that the use of a radioactive phantom filled with long-lived decaying isotopes could reduce sources of error such as dose calibrator variability and site-specific physicist hands-on training and experiences.

Encouraged by the above evidence, we aimed in this research to evaluate the relevance of mixing quantitative results coming from multisite PET exams among European facilities involved in a multi-center clinical trial. The QUICK project (Quantitation Unified Intercomparison Control Kit) was launched with the purpose of evaluating the impact of clinical protocol differences over scanner introduced variability. We realize the variability introduced with different scanner types is the ultimate goal for such a comparison, but we focus on protocol variability impact first as the initial step to understand the larger picture.

With the purpose of understanding the impact of different scanner operators, the same acquisitions and reconstructions were done on each site using the QUICK phantom. This step was followed with a series of acquisitions and reconstructions using local setup preferences as well as the site’s recommendations for clinical reconstruction.

The final statistical analysis of all data provided by the sites was expected to point out the impact of sites’ clinical practice on absolute quantitation in terms of contrast recovery, background variability, and lung error.

Results obtained using optimized protocols should be compared within the different participating sites to evaluate the global achievable accuracy of the multi-center clinical trials.

In summary, the aim of this study was to estimate the global statistical multi-center quantitation accuracy taking into account the discrepancies between participating PET facilities, using a solid phantom with fixed and accurately known activities and volumes.

The auxiliary aim of this collaborative work was to evaluate the impact of BSREM reconstruction on quantitation reliability, which is a unique feature of GE’s D-IQ PET-CT scanner selected for this multi-center study.

## Materials and methods

All the participating sites were equipped with a GE Discovery IQ PET/CT system, which is a state-of-the-art bismuth germanate (BGO) PET. It offers very high sensitivity and, associated with a new regularization algorithm (BSREM) named Q.Clear [11], raises an opportunity to get more accurate quantitation. Moreover, its high sensitivity allows to redefine dose prescription and exam durations.

The acquisitions were performed by medical physicists on fully calibrated PET/CT scanners.

In order to evaluate quantitation performance, the NEMA-defined parameters contrast recovery (CR) and background variability (BV) were collected and analyzed [12–15].

In the BSREM reconstruction algorithm, the noise penalty function strength named “Beta ” (*β*) is directly correlated with CR and BV. Consequently, it is relevant to this study to keep track of the site’s preference in *β* value selection. This penalty term provides level of regularization based on the difference between neighboring voxels as well as their sum.

A solid ^68^Ge phantom (QUICK phantom) based on IEC61675/NEMA Body IQ phantom geometry and activity concentrations was manufactured for this study allowing a better identification of the eventual causes of quantitative discrepancies among the participating sites.

In order to avoid discrepancies related to data analysis in each participating site, it was decided to use the built-in software provided by GE Healthcare and available on operating console to perform NEMA NU-2 2012 image quality test.

### ^18^F-FDG phantom acquisition

**Fig. 1:**
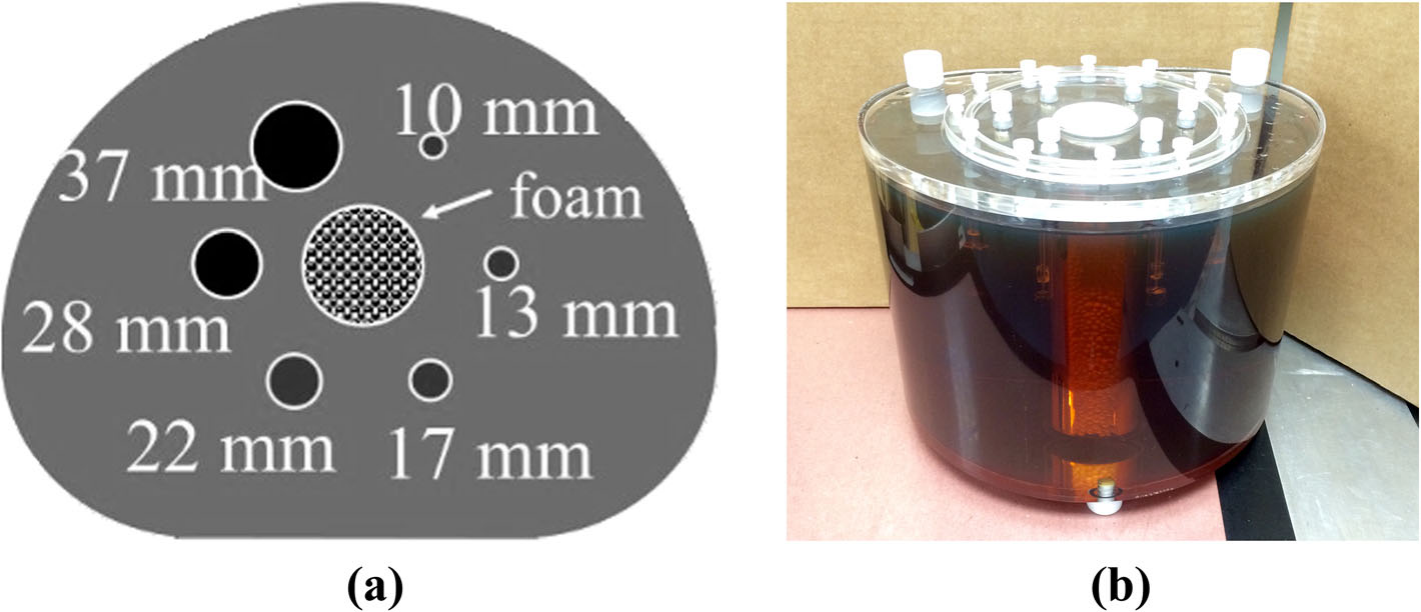
**a** Scheme of the IEC61675/NEMA NU2-2012 Image Quality Body Phantom (in black the cold spheres and in gray the hot spheres) and **b** a picture of the QUICK phantom

In the first part of the study, data from standard NEMA image quality test with an ^18^F-FDG phantom from seventeen different sites across Europe were combined. Each of the participating sites followed the manufacturer-provided protocol conducting three acquisitions with the IEC61675/NEMA NU2-2012 PET Image Quality Body Phantom. This phantom is a torso-like cavity containing 6 hollow spheres (internal diameters of 10, 13, 17, 22, 28, and 37 mm) surrounding a lung insert. The phantom was filled on each site with an ^18^F-FDG solution of approximately 53 MBq at the start of acquisition (Fig. 1a). The four smallest spheres were filled keeping sphere (21.2 kBq/ml) to background (5.3 kBq/mL) ratio of 4:1, while the largest two spheres were filled with water. The lung insert was filled with a non-radioactive mix of water and Styrofoam beads.

According to the NEMA NU2-2012 image quality test, there was also out-of-field activity (120 MBq) in a line source in the scatter-fraction phantom.

The acquisition and reconstruction parameters for the NEMA procedure, followed during the ^18^F-FDG phantom tests, are listed in Table 1.

**Table 1:** Acquisition and reconstruction parameters for the standard NEMA test with ^18^F-FDG phantom

**CT parameters**	**PET parameters**
**Scan type:** helical, full	**Scan time:** 5 min 52 s, 6 min 06 s, 6 min 21 s
**Tube voltage:** 120 kV	**Matrix size:** 256 × 256
**Tube rotation:** 0.5 s	**DFOV:** 40 cm
Auto mA modulation	**Attenuation correction type:** CT
**Slice thickness:** 3.75 mm	**Recon type:** VPHD
**DFOV:** 70 cm	**Iterations:** 8
	**Subsets:**12
	**Post-filter cutoff:** 2 mm
	***Z*-axis filter:** none

### QUICK phantom acquisition

Five^1^ out of the seventeen sites performed acquisitions with the QUICK solid radioactive phantom (^68^Ge) (Fig. 1b) that was designed and manufactured by Eckert & Ziegler^2^ to meet the IEC61675/NEMA NU2-2012 image quality body phantom features (6 spheres and a lung insert). To meet the requirements of this standard procedure, the total ^68^Ge activity in the phantom was 63.94 MBq at calibration, allowing a relatively easy shipment from one site to another. The two biggest spheres (28 and 37 mm in diameter) were made of cold resin (non-radioactive) and the four smallest ones with ^68^Ge resin at a concentration of 25.2 kBq/mL. The background activity concentration was at 6.36 kBq/mL so that the ratio of spheres to background was 3.96:1. Contrary to the NEMA NU2-2012 image quality test, there was no out-of-field activity during the acquisitions to avoid any unexpected effect of external source positioning.

The five centers involved in the QUICK phantom acquisition are identified in this paper as QUICK sites 1–5.

The data acquisition involved 5 measurements per day for a span of 5 days to account for the effect of the Poisson statistics and variability in phantom positioning, respectively. Thus, a dataset of 25 images have been used for this analysis for each site. Table 2 summarizes the acquisition and reconstruction parameters for the different tests.

**Table 2:** Summary of the test format (overview) realized with the QUICK phantom in each QUICK site. The rows denote the acquisition parameters (NEMA protocol/clinical protocol for the particular site), and the columns represent the reconstruction parameters (NEMA protocol/clinical protocol for the particular site) used in the test

		Reconstruction parameters			
		NEMA		Clinical	
		OSEM	BSREM (***β*** = 25)	OSEM	BSREM
**Acquisition parameters**	**NEMA**	**Test 1,** 25 datasets	**Test 2,** 25 datasets	**Test 3,** 25 datasets	**Test 4,** 25 datasets
	**Clinical**			**Test 5,** 25 datasets	**Test 6,** 25 datasets

In test 1, the QUICK phantom was scanned with the acquisition and reconstruction parameters recommended by the manufacturer for the standard NEMA NU2-2012 procedure (same as in Table 1 for the ^18^F-FDG phantom). The NEMA-IQ protocol recommended by the manufacturer does not include PSF because not all users have access to PSF, whereas a user with access to BSREM automatically includes access to PSF.

In test 2, the RAW data was re-processed using the BSREM algorithm and a *β* value of 25 optimized to match the background noise level (BV) observed in the corresponding OSEM reconstruction (test 1). Increasing beta value results in smoother background (preferred by physicians) at the expense of reduced contrast recovery [16]. This avenue was not explored as the intent of the paper is to compare site to site variations, not the impact of different beta values in clinical images.

In test 3 and test 4, each QUICK site re-processed the same RAW data using their own clinical reconstruction parameters optimized for OSEM and BSREM algorithms, respectively. For OSEM, the number of iterations for clinical reconstruction was the site’s choice as preferred by the site’s reading physicians and is a compromise between signal recovery and background noise level. The number of iterations for BSREM (25 iterations) is selected by the manufacturer until convergence is reached as intrinsically established by the BSREM algorithm (Table 2).

In test 5 and test 6, the QUICK phantom was re-scanned in each site with its own clinical acquisition protocol (in terms of FOV (field of view), scan time, and acquisition matrix) and re-processed with clinically optimized OSEM and BSREM algorithms already used in test 3 and test 4, respectively.

Detailed acquisition and reconstruction parameters for each test are shown in Table 3.

**Table 3:** Acquisition and reconstruction parameters for each test

	**Test 1**	**Test 3**	**Test 5**
**Matrix size**	256	256	256
**FOV (cm)**	40	40–70	40–70
**Reconstruction algorithm**	OSEM	OSEM	OSEM
**Iterations**	8	4–6	4–6
**Subsets**	12	12	12
**Filter (FWHM)**	2	4.8–6.4	4.8–6.4
***Z*-filter**	None	Standard (weight 1 4 1)	Standard (weight 1 4 1)
**Corrections**	CTAC, scatter, randoms	CTAC, scatter, randoms	CTAC, scatter, randoms
**PSF correction**	No	Yes/no	Yes/no
	**Test 2**	**Test 4**	**Test 6**
**Matrix size**	256	256	256
**FOV (cm)**	40	40–70	40–70
**Reconstruction algorithm**	BSREM	BSREM	BSREM
**Iterations**	25	25	25
***β***	25	350	350
**Corrections**	CTAC, scatter, randoms	CTAC, scatter, randoms	CTAC, scatter, randoms
**PSF correction**	Yes	Yes	Yes

In each site, the acquisition time for the QUICK phantom was adjusted to compensate for the decay of ^68^Ge activity over time to meet the NEMA NU2-2012 (test 1 to test 4) or to meet the clinical protocol (test 5 and test 6) count requirements.

### Data analysis

For a consistent analysis, all the datasets were analyzed by each site using the automated GE NEMA image quality analysis software available on the operating console: CR and BV for each sphere in the phantom were calculated using each reconstruction method. Similarly, the LE was also determined using each reconstructed method. These variables are defined in the NEMA standards publication NU2-2012 image quality test [17].

## Results

### ^18^F-FDG phantom acquisition

**Fig. 2:**
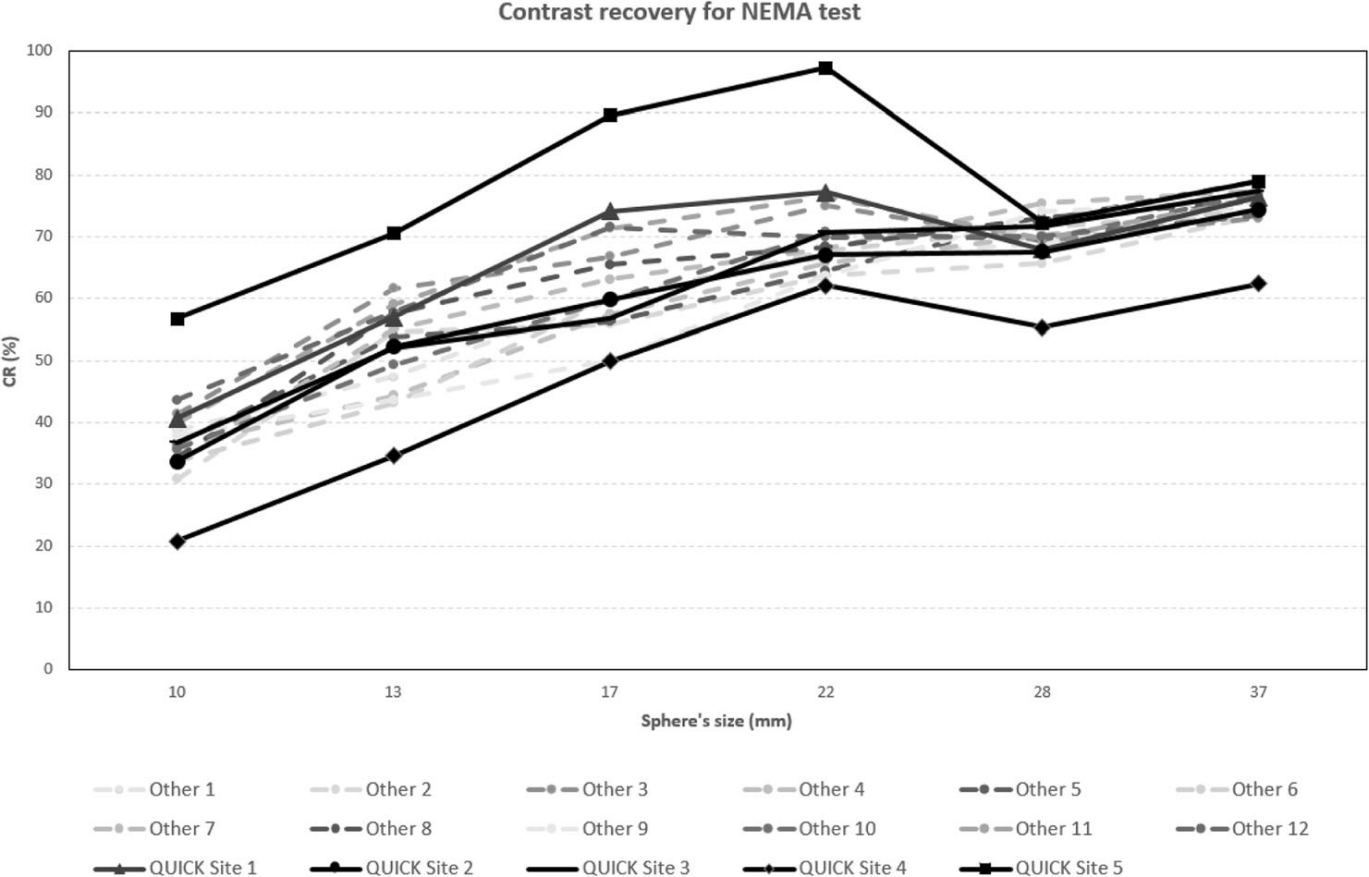
CR (%) values for the standard NEMA test performed with ^18^F-FDG phantom

**Fig. 3:**
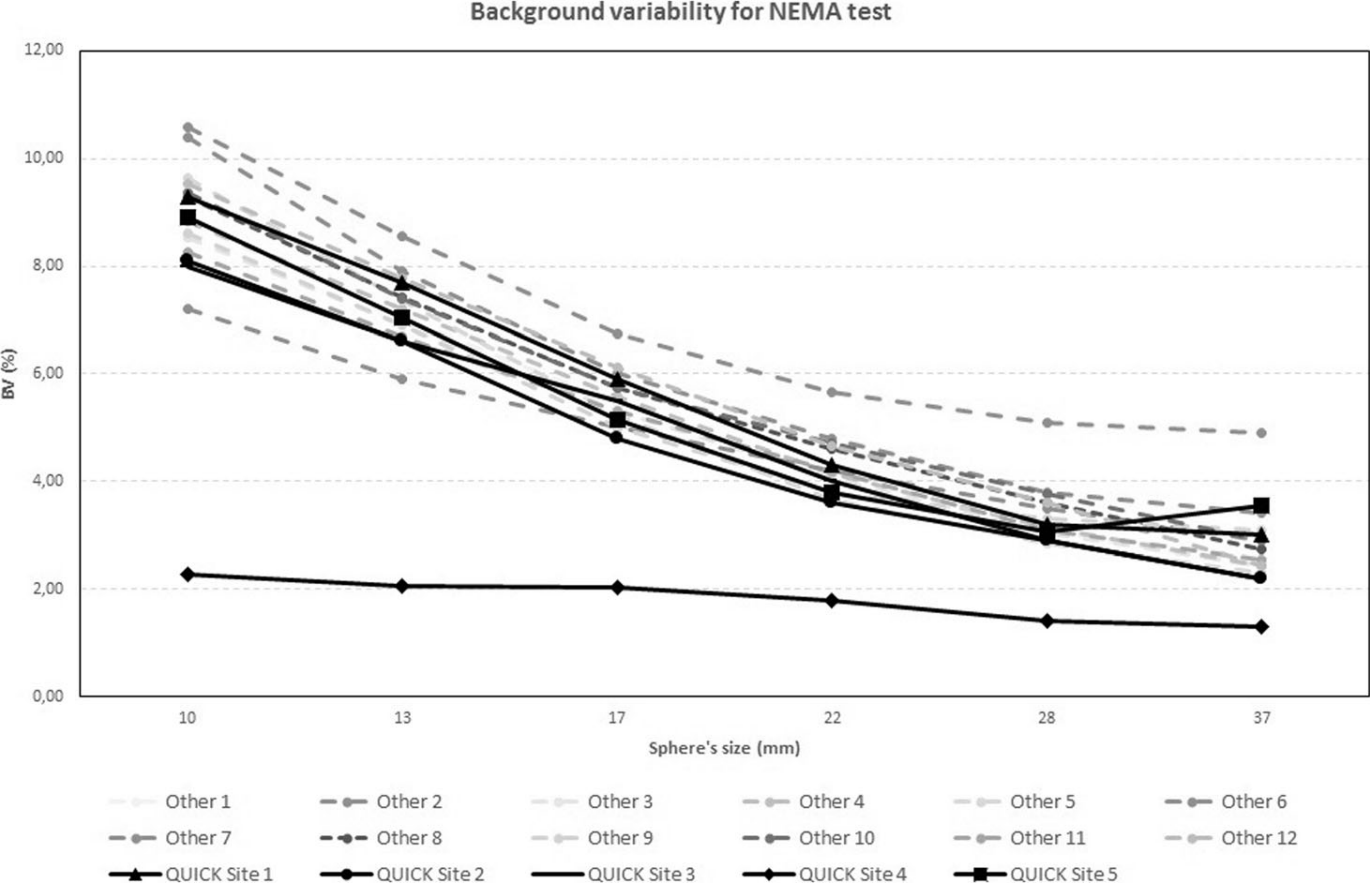
BV (%) values for the standard NEMA test performed with an ^18^F-FDG phantom

**Fig. 4:**
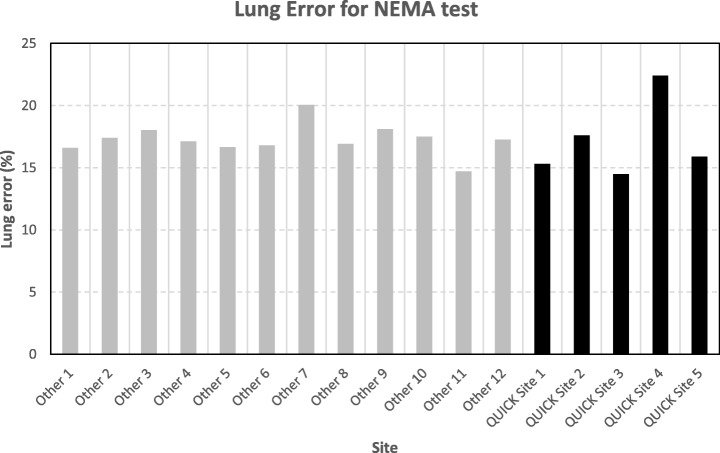
Lung error (LE in %) values for the standard NEMA test performed with ^18^F-FDG phantom

The results of the NEMA NU2-2012 image quality test, in terms of CR, BV, and LE, are presented in Fig. 2, Fig. 3, and Fig. 4, respectively.

As expected, the majority of participating centers obtained similar results, and only two centers (QUICK Site 4 and QUICK site 5) showed unexpected data.

Excluding these 2 centers, the CR values range from 30.8–43.6 to 63.7–77.2 from the smallest to the largest of the hot spheres, respectively. For the cold spheres (28 mm and 37 mm), the same parameter ranges from 65.7–75.4 to 73–79.1.

### QUICK phantom acquisition

**Fig. 5:**
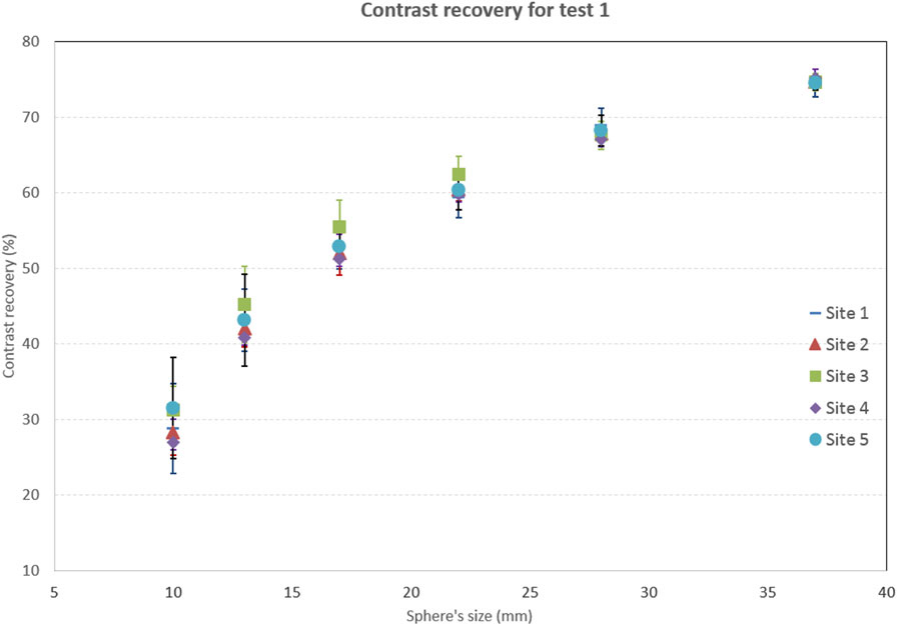
Contrast recovery and background variability for test 1 for the QUICK sites

**Fig. 6:**
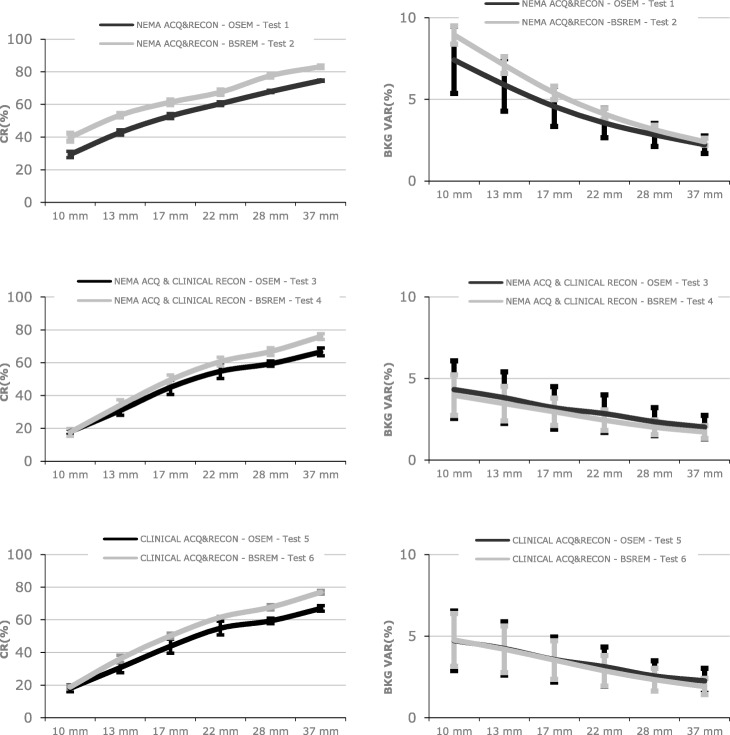
CR (left column) and BV (right column). Top row, tests 1 and 2. Middle row, tests 3 and 4. Bottom row, tests 5 and 6. All tests were performed with the QUICK phantom

**Fig. 7:**
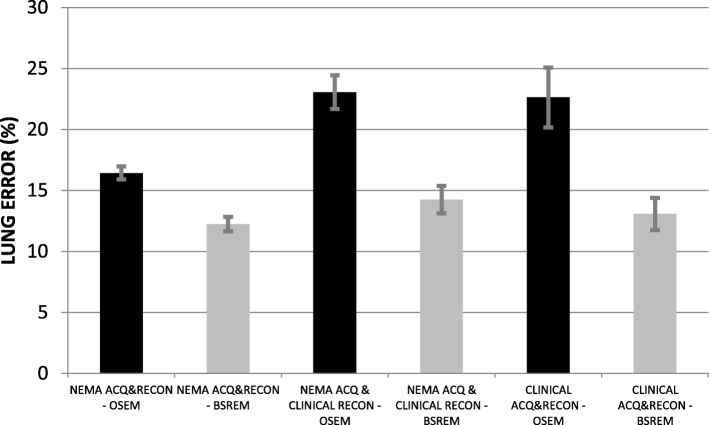
LE values for the six tests performed with the QUICK phantom

The results of the six QUICK phantom tests, as average and standard deviation of acquisitions among all QUICK sites, are shown in Table 4 (Figure 5 for graphic plot) and Fig. 6 and Fig. 7.

**Table 4:** CR, BV, and LE for the six tests performed with QUICK phantom as average ± standard deviation of datasets from all QUICK sites combined [17]

	NEMA ACQ and RECON OSEM (test 1)	NEMA ACQ and RECON BSREM (test 2)	NEMA ACQ and clinical RECON OSEM (test 3)	NEMA ACQ and clinical RECON BSREM (test 4)	Clinical ACQ and RECON OSEM (test 5)	Clinical ACQ and RECON BSREM (test 6)
Sphere	**Contrast recovery (%): average ± SD**					
10 mm	29.4 ± 2.0	39.9 ± 2.5	17.9 ± 1.6	17.6 ± 2.4	18.0 ± 2.1	18.8 ± 1.1
13 mm	42.9 ± 1.6	53.5 ± 1.1	30.8 ± 3.0	34.1 ± 3.4	30.7 ± 3.0	36.1 ± 2.2
17 mm	52.9 ± 1.6	61.4 ± 1.5	45.0 ± 4.3	49.5 ± 3.1	43.8 ± 4.4	50.2 ± 1.6
22 mm	60.5 ± 1.2	67.5 ± 1.4	54.8 ± 4.5	60.7 ± 2.6	54.9 ± 4.3	61.6 ± 0.5
28 mm	67.9 ± 0.7	77.7 ± 1.0	59.3 ± 1.6	66.8 ± 2.4	59.3 ± 1.7	67.5 ± 1.5
37 mm	74.6 ± 0.4	83.1 ± 0.6	66.6 ± 2.3	75.9 ± 1.8	66.9 ± 1.7	76.9 ± 1.1
Sphere	**Background variability (%): average ± SD**					
10 mm	7.4 ± 2.1	8.9 ± 0.6	4.3 ± 1.8	4.0 ± 1.2	4.7 ± 1.8	4.8 ± 1.6
13 mm	5.9 ± 1.6	7.1 ± 0.5	3.8 ± 1.6	3.5 ± 1.1	4.2 ± 1.6	4.2 ± 1.4
17 mm	4.6 ± 1.2	5.4 ± 0.4	3.2 ± 1.3	3.0 ± 0.8	3.6 ± 1.4	3.5 ± 1.2
22 mm	3.6 ± 0.9	4.1 ± 0.3	2.8 ± 1.1	2.4 ± 0.6	3.1 ± 1.2	2.9 ± 0.9
28 mm	2.8 ± 0.7	3.1 ± 0.3	2.3 ± 0.9	2.0 ± 0.4	2.6 ± 0.9	2.3 ± 0.7
37 mm	2.2 ± 0.5	2.4 ± 0.2	2.0 ± 0.7	1.7 ± 0.4	2.3 ± 0.8	1.9 ± 0.5
	**Lung error (%): average ± SD**					
	16.4 ± 0.5	12.2 ± 0.6	23.1 ± 1.4	14.3 ± 1.1	22.6 ± 2.5	13.1 ± 1.3

## Discussion

The most often recommended procedure to evaluate the quantitation variability among PET facilities is to perform acquisitions and reconstruction of a fillable image quality phantom using the local setting of parameters and to extract quantitative data from resulting images. Following suit, the first part of this project showed that the variability introduced in the filling of the ^18^F-filled NEMA-IQ phantoms, scanned and imaged following same protocols across sites, is larger than the one from a ^68^Ge filled phantom where each site was allowed to select the acquisition and reconstruction protocols. The main goal of limiting to a single scanner type (D-IQ from GE) was to control all other parameters in this comparison except for the imaging protocol selection between sites. This conclusion is further supported by the small variability observed when same imaging protocol is used with the QUICK phantom.

In this study, we have tried to assess and demonstrate the possibility of performing quantitative multisite clinical trials in PET using QUICK phantom. In addition, we also made an effort to understand the impact and potential causes of discrepancies among different sites if any.

In order to avoid additional possible causes of discrepancies, we decided to extract quantitative data using the automatic processing available on the scanner’s console.

As mentioned above, two centers of the 17 sites (QUICK site 4 and QUICK site 5) showed unexpected data from the ^18^FDG phantom, despite having detailed directions for filling and imaging of the phantom. Their results can be explained by placing a larger than prescribed ratio of activity concentration between spheres and background (QUICK site 5) and too high activity in the background above the NEMA prescribed value (QUICK site 4).

These results clearly demonstrate the possible issues when trying to compare systems using ^18^F-FDG fillable phantom due to a different level of experience of the operators (e.g., handling of FDG, accurate filling of the spheres, careful alignment of the phantom). A solid radioactive phantom filled with ^68^Ge can be used to avoid such errors [10], and the reproducibility of the measurements can thus be enhanced.

Results of the standard NEMA test using the QUICK phantom support this assessment by showing more consistent results.

With equal acquisition conditions among sites, the BSREM algorithm gives better or similar results than the OSEM algorithm, i.e., higher CR, lower or similar BV, and a lower LE. The exceptions are due to the specific selection of *β* value (25) was intended to produce comparable background variability in the BSREM images with respect to OSEM images. This selection was not optimal, particularly for smaller ROIs (Fig. 5 top right), highlighting the trade between beta selection and CR. Larger clinical beta values (e.g., 350) produce smoother images at the expense of CR.

In general, all the tests performed using the 68-Ge QUICK phantom led to small variability of the CR which is the most relevant parameter in the perspective of quantitative PET studies. One aspect of the results presented here, that is “the ^68^Ge NEMA-IQ standard NEMA-2012 test”, provides a unique opportunity to observe the variability introduced from scanner to scanner when most other variables are confined to a very small change of their own.

Focusing on test 6 (the site’s clinical acquisition and reconstruction parameters), BSREM algorithm reduces the CR variability among sites when compared with the OSEM algorithm. This result supports the use of BSREM as the preferred protocol in a multi-center clinical trial regardless of the site-specific beta value. This may reduce the variability introduced by patient size and body habitus [18].

Logistical challenges in transporting the QUICK phantom from site to site (e.g., custom approvals, local regulations with respect to transport and handling of radioactive material), resulted in delays of sites performing the measurements. Hence, each site performed the measurements with different total activity of the phantom. We compensated the decaying activity through longer acquisition times. However, it should be noticed that the acquisition conditions are not fully equivalent as the ratio of true events to random events does not remain constant with changing activity.

The QUICK project was designed as a phantom study for comparing reconstruction protocols and selection of parameters. Hence, it does not take into account other parameters like dose calibrator, injected activity, serum glucose level, and post-injection time. To minimize variability of these parameters, multi-center participants must meet accreditation requirements that should include, for instance, the use of traceable sources for calibration of their dose calibrator, and similar patient preparation [7, 19].

## Conclusion

This study demonstrates the variability introduced by using different imaging protocols is smaller than the variability introduced by the operator when a NEMA-IQ phantom is handled and imaged by different sites following identical protocols. In turn, this demonstrates that the use of phantoms like the NEMA-IQ phantom for multi-center evolution of protocols is unreliable.

BSREM algorithm reduces the variability of contrast recovery in PET images compared to the OSEM algorithm in different clinical acquisition conditions and reconstruction parameters across multiple centers. In the future, we plan to study the impact of the patient’s preparation on the variability of these measurements and to evaluate if harmonization of the injected dose is a necessary requirement for this kind of comparison studies.

## Data Availability

The datasets used and/or analyzed during the current study are available from the corresponding author on reasonable request.

## Footnotes

1 Institut Universitaire du Cancer de Toulouse (France), ASST Monza (Italy), Affidea Wroclaw 3 (Poland), Affidea Poznan 1 (Poland), and Hospital Universitari de Bellvitge—IDIBELL (Spain)

2 Eckert & Ziegler Isotope Products, Inc., 24937 Avenue Tibbitts, Valencia, CA 91355
